# Clinical characteristics and management of functional neurological disorders (FND) mimicking stroke in emergency settings: a functional stroke mimic cases

**DOI:** 10.3389/fneur.2024.1461320

**Published:** 2024-09-04

**Authors:** Paola Caruso, Yvonne Radin, Laura Mancinelli, Magda Quagliotto, Tiziana Lombardo, Stefania Pavan, Mauro Catalan, Andrea Clarici, Matteo Bulfon, Alberto Benussi, Paolo Manganotti

**Affiliations:** ^1^Department of Medical, Surgical and Health Sciences, Cattinara Hospital, University of Trieste, Trieste, Italy; ^2^Department of Pediatrics, Neurology Clinic, Institute for Maternal and Child Health Burlo Garofolo, Trieste, Italy

**Keywords:** stroke mimic, functional neurological disorders, thrombolysis, emergency department, motor disorder

## Abstract

**Background:**

FNDs mimicking a stroke represent a growing challenge in the emergency department (ED). A comprehensive diagnostic approach involving clinical evaluation and neuroimaging is essential to differentiate stroke from mimics. The safety profile of thrombolysis justifies its use where FNDs cannot be ruled out. This approach highlights the need for more precise diagnostic tools and protocols to improve patient care and reduce unnecessary treatments. Distinguishing FNDs from actual cerebrovascular events is critical yet difficult, particularly under time constraints. Given the urgency and potential severity of strokes, intravenous thrombolysis is frequently administered even when FNDs cannot be definitively excluded.

**Methods:**

This retrospective study analyzed data of participants admitted to the Trieste University Hospital Stroke Unit between January 2018 and December 2022, focusing on those presenting with sudden-onset focal neurological deficits mimicking a stroke, with some presenting within the reperfusion treatment window (<4.5 h from symptoms onset). We obtained detailed clinical evaluations and neuroimaging, and administered thrombolytic therapy in selected cases.

**Results and discussion:**

We included 84 participants presenting with stroke mimics (average age of 45 yo) predominantly female (65.5%). Most common presentations: hemiparesis or hemisensory loss (75%), speech disorder (10.7%), vertigo/gait disorders (4.8%). History of psychiatric disorders was found in 32.1% of cases, and 48.8% had prior neurological disease or stroke risk factors. Advanced neuroimaging was performed in 43 cases yielding normal or non-specific results. Thrombolysis was safely administered in 31%. Patients mostly recovered within the first 24 h from admission (44.7%). We compared this FND’s sample with 291 patients with mild ischemic stroke (NIHSS ≤7).

## Introduction

FNDs present a complex epidemiological landscape, historically complicated by ambiguous definitions. Recent advancements, particularly the DSM-5 (2013) revisions, have helped clarify this by defining FNDs based on the presence of physical symptoms that are inconsistent with neurologic disease, emphasizing that psychopathology is not a necessary component for diagnosis.

Conversion disorder, a subset of FNDs, is reported in 20–25% of patients in a general hospitals. Somatic symptom disorders or medically unexplained symptoms occur in 10–20% of middle-aged individuals (50–65 years) and 1.5–13% of those aged 65 years or older. In emergency departments (ED), FNDs ranks as the second most common reason for outpatient neurology consultations, following headaches (1). Affects both sexes and all age groups, FNDs nonetheless exhibits a higher prevalence among women, particularly between 35 and 50 years old, who constitute 60–75% of the patient population (2, 3).

Recognition of stroke mimics can be challenging especially in the acute setting (4). Stroke mimics (SM) comprise both non-neurological conditions (such as hypertension, diabetes, metabolic dysfunction, cardiac diseases, malignancy) and neurological disorders (including seizures, migraine, meningitis). Notably, about 30% of patients with FNDs are admitted to the ED with symptoms mimicking a stroke (5). Differentiating these SM from actual strokes poses a significant clinical dilemma, as timely administration of thrombolytic therapy, a standard treatment for ischemic stroke, may be dangerous or unnecessary if not strictly necessary as in SM cases (6, 7).

The prevalence of stroke mimics varies across clinical settings and patient populations. Misdiagnosis can lead to delayed treatment, unnecessary healthcare costs, and increased burden on healthcare resources (8). In recent years, there has been growing interest in the study of stroke mimics, driven by advances in neuroimaging, increased awareness among healthcare providers, and the need to optimize resource allocation in stroke care (9, 10). In a previous study, we addressed the complexities of managing these functional disorders in stroke emergency departments (9).

FNDs can cause significant distress and disability, representing a critical condition requiring prompt diagnosis and accurate follow-up to prevent acute recurrences and misdiagnosis in the ED.

This study aims to analyze the incidence and recurrences rates of SM due to FNDs in the ED, and to discuss the management decisions regarding thrombolytic therapy in FNDs cases. We also delve into the pathophysiology of this specific and pleomorphic functional disorder in the emergency context. By gaining a deeper understanding of SM, healthcare professionals can enhance their diagnostic accuracy and provide more appropriate and tailored care to patients, ultimately leading to improved clinical outcomes.

## Materials and methods

### Participants

This retrospective study spanned 5 years, from January 1, 2018, to December 31, 2022. We examined data of participants admitted to the ED of Cattinara Hospital, University of Trieste, presenting with acute neurological deficits initially suggestive of stroke but later diagnosed as FNDs. In the modeling of control group, data were collected from 291 patients admitted for mild-to-moderate ischemic stroke (NIHSS≤7) from January 1, 2020 to December 31, 2022. Inclusion criteria for the control group were: ischemic stroke with mild symptoms (NIHSS≤7), age 18–75 yo, arrived in our ED for a sudden neurological deficit (within 4.5 h, eligible for reperfusive treatment) and admitted in stroke unit. Exclusion criteria: history of previous cognitive impairment, previous mRS ≥ 2.

**Figure 1:**
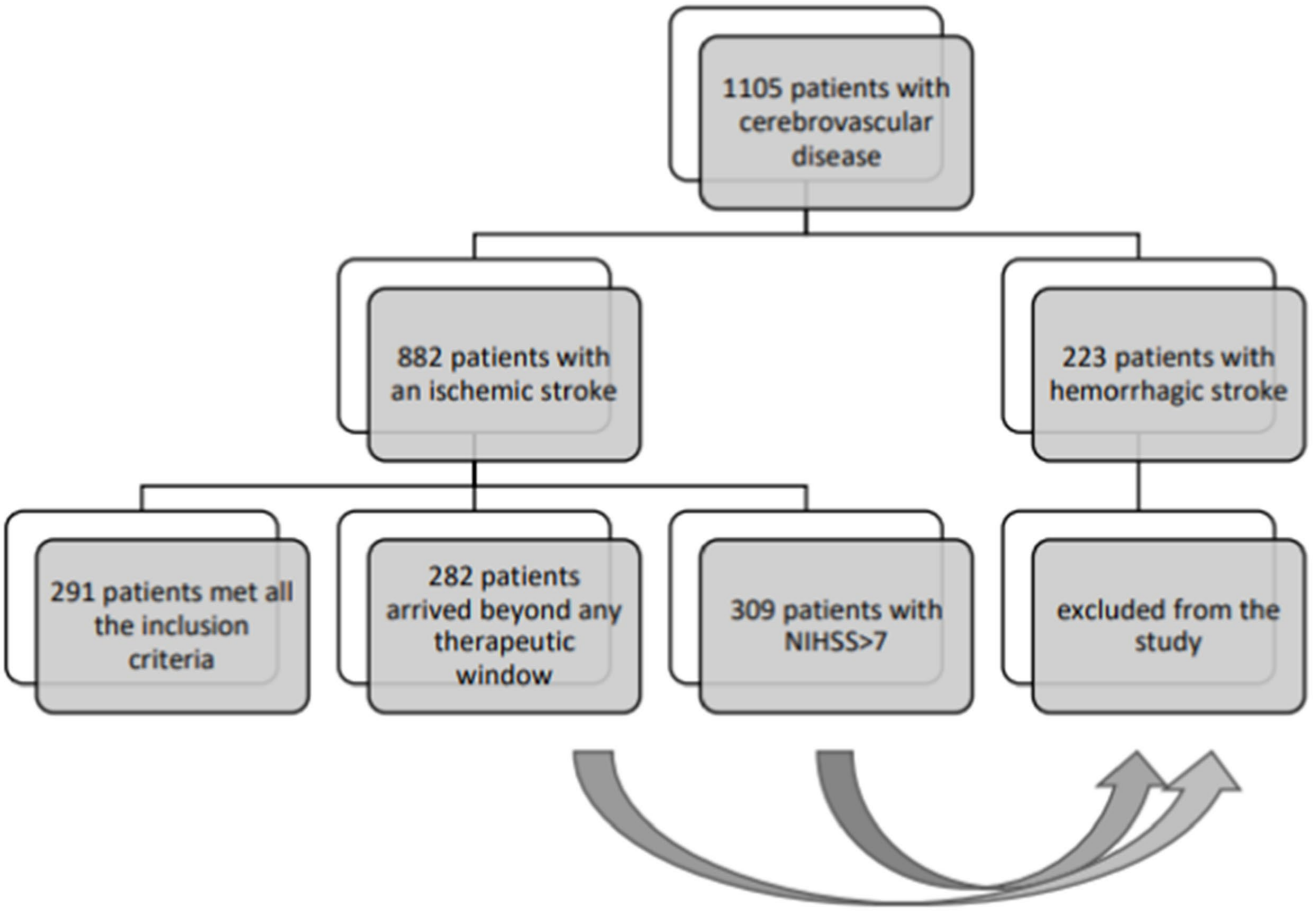
Sample selection flow chart.

Annually, approximately 600 patients with acute stroke are admitted to our neurological Stroke Unit. These cases are typically admitted in the ED under the “stroke protocol,” activated for patients with acute, datable, focal neurological symptoms, where physicians have a 4.5 h window from symptom onset to administer reperfusion therapy (data are reported in Figure 1). After initial ED admission, patients undergo neuroimaging before being transferred to the Stroke Unit.

We documented patient details including demographics, educational level, stroke risk factors, and medical history. Initial symptoms and concurrent pathologies were also recorded. Within this cohort, we explored various aspects, including thrombolysis administration, symptom presentation and duration, and sex-based differences in SM diagnoses. Clinical evaluations were conducted at admission and after 3 months to note any recurrences.

### Neuroimaging

Brain CT scans and, in selected cases, advanced neuroimaging were performed using a Brilliance iCT 256-slice scanner (Philips Medical Systems, Best, Netherlands). The CT perfusion acquisition protocol entailed injecting 75 mL of contrast medium, followed by a 40 mL saline bolus, both at a rate of 4 mL/s. This was complemented by three dimensional axial acquisitions covering the entire brain volume, with slice reconstruction set to 5 mm, facilitated by a series of repeated scanner table movements. These acquisitions were executed at a 4-s interval, resulting in a total scanning duration of 60 s. The brain perfusion software operates on the central volume principle, employing a closed form, non-iterative deconvolution method to evaluate the mean transit time (MTT). Areas below the density/time curves are used to determine the cerebral blood volume (CBV). Cerebral blood flow (CBF) maps are derived by calculating the ratio of CBV to MTT.

### EEG recordings

EEG was performed using a Be Plus PRO amplifier EEG system (EB NEURO, Florence, Italy) and was continuously recorded from 19 scalp sites positioned according to the 10–20 International System. The ground electrode was positioned in Fpz, while the reference one was positioned at Cz. Skin/electrode impedance was maintained below 5 kΩ, and the sampling rate was set to 128 Hz. EEG signals were filtered by second-order band-pass Butterworth filter with 0.1–30 Hz cut-off frequencies, and the epochs containing artifacts were discarded. Brain oscillatory activities were assessed by standard clinical qualitative visual inspection of EEG tracings by experienced neurologists, in order to identify clinically significant epileptiform patterns and EEG rhythm alterations. Our diagnostic protocol for managing acute cerebrovascular pathology includes performing an EEG on all patients with suspected stroke, in order to identify any severe epileptic activity.

### Statistical analysis

For the basic statistical analysis comparing stroke mimic and ischemic patients, we performed descriptive statistics and statistical tests to determine significant differences between the two groups. The statistical analyses included calculations of counts and relative frequencies for categorical variables. Chi-Square test was used to determine the statistical significance of differences in categorical variables between the two groups.

**Figure 2:**
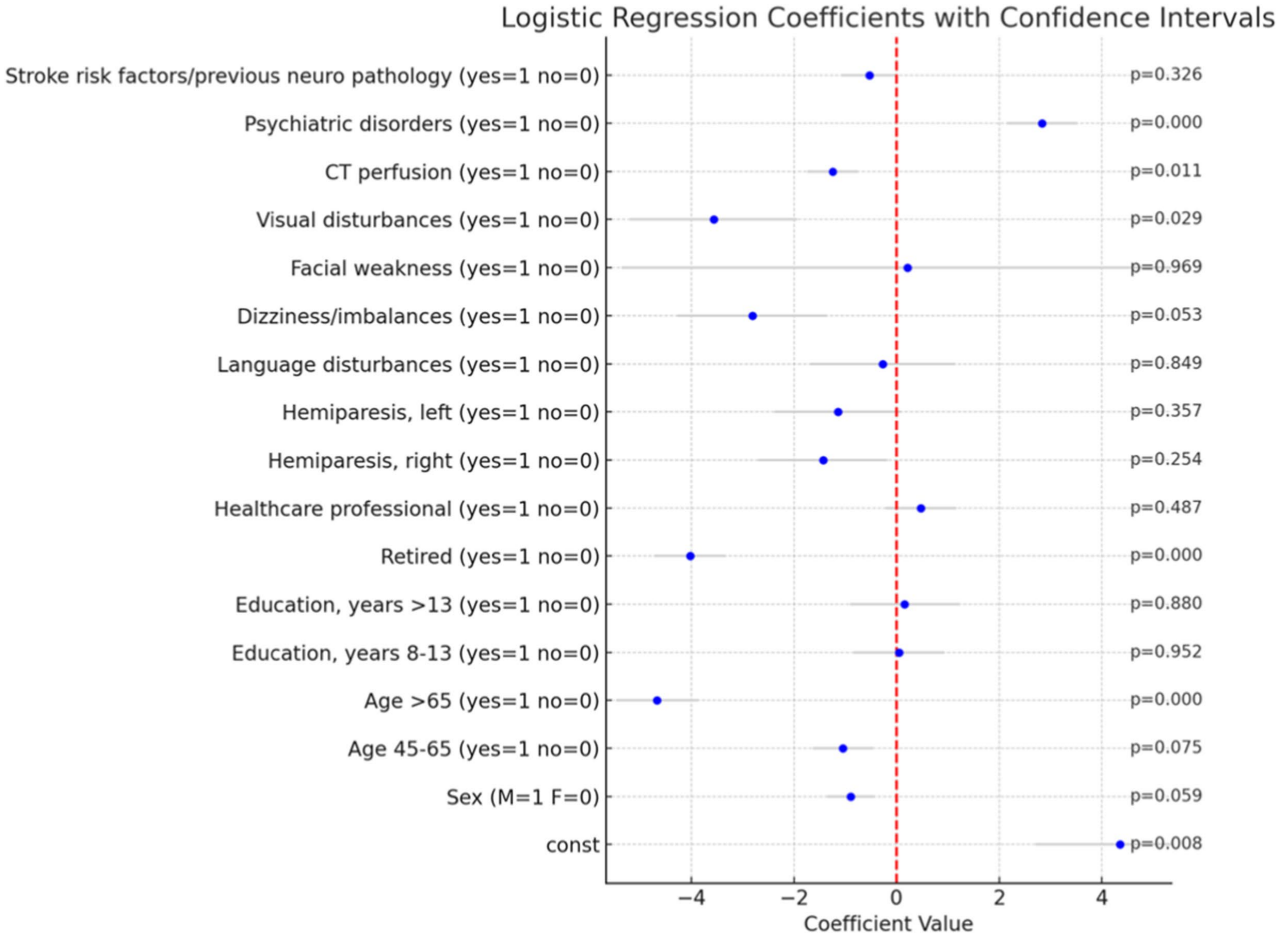
Logistic regression coefficients with confidence intervals and *p*-value. The bar plot illustrates the coefficients of the logistic regression analysis performed to predict the likelihood of a Mimic diagnosis versus an Ischemic diagnosis. Each bar represents the coefficient of a variable, and the gray lines indicate the 95% confidence intervals for these coefficients. *p*-values are reported on the right.

The analysis was performed using a dataset that included several predictor variables, which were dummy coded to enhance the performance of multivariate analyses. To ensure robustness and completeness of the data, we employed the Multiple Imputation by Chained Equations (MICE) technique to handle missing values, followed by logistic regression. The analysis was performed using the stats models library in Python. The results are summarized in Figure 2.

## Results

Our study cohort consisted of 84 participants, with females comprising 65.0% (55 patients) of the group. The average age was 46.7 ± 14.7 years. The most common presentation was hemiparesis (75.1%), with a notable distinction between right-sided (27.4%) and left-sided (47.6%) manifestations. Other presentations included language disturbances (10.7%), posterior circulation disturbances such as imbalance and dizziness (4.8%). Additionally, facial weakness was observed in 1 participant, visual disturbances in 1, and 6 participants exhibited multiple symptoms (see Table 1).

**Table 1:** Baseline demographic and clinical characteristics of included participants.

Variable	Mimic (*N* = 84)	Ischemic (*N* = 291)	*p*-value*
Sex (male)	29 (34.5%)	169 (58.1%)	< 0.001
Age, years			
<45	32 (38.1%)	15 (5.2%)	< 0.001
45–65	45 (53.6%)	64 (22%)	< 0.001
>65	7 (8.3%)	212 (72.9%)	< 0.001
Education, years			
<8	2 (2.4%)	47 (16.2%)	0.008
8–13	52 (61.9%)	195 (67%)	0.150
>13	11 (13.1%)	36 (12.4%)	0.523
Missing data	19 (22.6%)	13 (4.5%)	
Retired	2 (2.4%)	191 (65.6%)	< 0.001
Missing data	14 (16.7%)	8 (2.7%)	
Healthcare professional	14 (16.7%)	21 (7.2%)	0.003
Missing data	15 (17.9%)	8 (2.7%)	
Hemiparesis, right	23 (27.4%)	126 (43.3%)	0.012
Hemiparesis, left	40 (47.6%)	98 (33.7%)	0.028
Language disturbances	9 (10.7%)	15 (5.2%)	0.114
Dizziness/imbalances	4 (4.8%)	28 (9.6%)	0.245
Facial weakness	1 (1.2%)	3 (1.0%)	1.000
Visual disturbances	1 (1.2%)	21 (7.2%)	0.071
Multiple symptoms	6 (7.1%)	**	
CT perfusion	43 (51.2%)	199 (69.1%)	0.004
Psychiatric disorders	27 (32.1%)	18 (6.2%)	< 0.001
Stroke risk factors/previous neurological pathology	41 (48.8%)	260 (89.3%)	< 0.001
Intravenous thrombolysis	26 (31.0%)	150 (51.5%)***	0.002

Data are median (IQR) or n (%). n = number of patients. This table clarifies the meaning of both absolute and relative frequencies for each categorical variables to highlights the differences between the two groups of patients (Mimic and Ischemic). *These p-values were calculated using the Chi-squared test to assess the statistical significance (*p* < 0.05) of the difference between the “Mimic” and “Ischemic” groups for each variable. **The classification of “multiple symptoms” which are very often not identifiable in the context of a specific damaged brain area appears not to be applicable in patients with ischemic stroke (where instead in most cases the site of the lesion is well identifiable based on the neurological examination and the neuroimaging). ***150 patients underwent reperfusive treatment: 130 intravenous thrombolysis, 18 intravenous thrombolysis and mechanical thrombectomy, 3 mechanical thrombectomy alone. Mimic patients underwent thrombolysis only.

All patients underwent a direct brain CT scan in the ED. In 43 cases (51.2%), CT angiography and CTP were also performed. CT perfusion maps were negative in all cases, with nonspecific alterations, mainly due to movement artifacts, noted in some instances. EEGs were recorded in 60 (70.6%) participants, revealing normal traces in the majority, while rapid rhythms and nonspecific theta oscillations were identified in three patients. Thrombolysis was administered to 26 patients (31.0%) patients, with the remaining 58 (69.0%) not receiving it, including three who declined treatment.

In our sample, 32.1% had a history of psychiatric disorders (predominantly anxiety disorder and major depression), 48.8% had previous neurological disease or stroke risk factors. Common neurological conditions included migraine, peripheral palsy, transient ischemic attacks, while stroke risk factors ranged from smoking to hypertension, diabetes, dyslipidemia and extracranial carotid stenosis.

A subgroup analysis considered patients’ timing of ED arrival beyond the limits for reperfusion treatment, adherence to the “stroke protocol,” and receipt of systemic thrombolysis. No significant differences were found between groups in terms of cardiovascular risk factors, previous or current psychiatric pathology, or previous neurological pathology (primarily neuropathic diseases, migraines, transient ischemic attacks). Similarly, no differences in educational level or symptom duration were reported.

**Figure 3:**
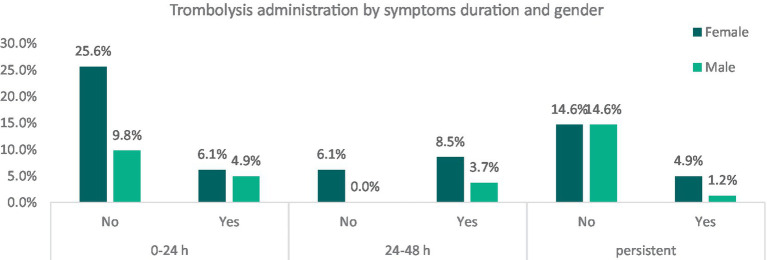
Relative frequencies of thrombolysis administration among patients, divided by gender and symptoms’ duration. It categorizes patients into males and females and further divides them based on whether they received thrombolysis treatment (“Yes” or “No”). These categories are then cross-referenced with the duration of symptoms. The graph illustrates the relative frequencies of thrombolysis administration among stroke mimic patients, segmented by gender and symptom duration. While this data highlights early intervention trends, it does not provide direct evidence of a placebo effect influencing symptom duration.

Symptom duration varied: 44.7% of patients experienced resolution within 24 h, 16.4% had symptoms lasting between 24 to 48 h, and 35.2% had persistent symptoms for days. Sex-based variations in symptom duration were observed, with shorter durations more prevalent among female participants (25.6% of female with standard treatment and 6.1% with reperfusive treatment showed symptoms resolution between 0 and 24 h) compared to males (9.8% of patients with standard treatment and 4.9% with thrombolysis) (Figure 3).

Symptoms recurrences were observed in 37% of cases, including 13 participants who underwent reperfusion treatment.

Regarding occupational activity at the time of the event we found that 16.7% of patients with FNDs miming a stroke were healthcare professionals, such as nurses or technicians [as already described in literature (11)].

We compared FNDs with 291 patients with ischemic stroke (NIHSS ≤7) admitted to our stroke unit.

In the multivariate logistic regression analysis several significant predictors of a Mimic diagnosis were identified, particularly within clinical and demographic factors. Among these, advanced age (specifically, patients older than 65 years) was strongly associated with a lower likelihood of a Mimic diagnosis (*p* < 0.001), as was retired status (*p* < 0.001). Conversely, the presence of psychiatric disorders (*p* < 0.001) significantly increased the likelihood of such a diagnosis. Additionally, visual disturbances were negatively associated with a Mimic diagnosis (*p* = 0.029).

To accurately assess the impact of age and education on the likelihood of a Mimic diagnosis, we employed a common statistical technique in regression analysis by omitting one category from each of these variables. Specifically, we excluded the <45 years category from the age variable and the <8 years category from the education variable. This approach was necessary to avoid the issue of multicollinearity, which arises when independent variables are highly correlated or mutually exclusive. In this case, each patient could only belong to one age group and one education level. By omitting one category, we created a reference group against which the effects of the other categories could be compared. This method allows for a clearer interpretation of the results, ensuring that the regression model provides meaningful and unbiased estimates of the impact of age and education on the likelihood of a Mimic diagnosis. These findings underscore the importance of considering both clinical and demographic factors in the differential diagnosis of Mimic conditions. The exclusion of specific categories from the age and education variables was crucial for maintaining the statistical integrity of the model and ensuring the validity of the results.

## Discussion

This study underscores the significant role of FNDs within stroke units, particularly among patients presenting with sudden weakness or speech disorder, which complicate emergency decision-making. It is crucial to promptly recognize FNDs mimicking a stroke to ensure appropriate treatment. However, the presence of positive signs does not always facilitate the quick exclusion of organic disease. In our sample, a significant number of SM who underwent thrombolytic therapy showed a long recovery from functional motor disorder. All patients had normal neuroimaging, even a considerable time after the acute presentation of functional symptoms. A large proportion had previous medical or neurological conditions, and many were employed in healthcare public-facing roles. Further studies are needed to determine if these factors predispose or precipitate this condition.

This data highlights the necessity of a multidimensional assessment for patients with FNDs, aiming to establish a strict follow-up and enhance psychotherapy. The observed prevalence of hemiparesis, commonly seen in FNDs, raises questions about its psychosomatic origin, aligning with existing literature. This is thought to result from the complex interplay of psychological stressors and altered brain function (12–14).

The integration of “Laterality in Somatization” by Min and Lee with Elliott D. Ross’s Emotion-type Hypothesis (ETH) provides a deeper understanding of the neurobiological basis of somatic symptom lateralization in emotional disorders. Min and Lee’s observation of somatic symptom lateralization emotional responses. The findings of somatic symptoms, particularly on the left side, indicate significant involvement of the right hemisphere, which predominantly processes emotional responses (15). Ross’s concept that primary emotions, often negative and linked to survival instincts, are predominantly processed by the right hemisphere, whereas social emotions, which are acquired and culturally modulated, are processed by the left hemisphere, offer insight into the lateralization of somatic symptoms (16). In our sample, a significant number of patients exhibited left-sided somatic symptoms, aligning with the hypothesis of the right hemisphere’s involvement in processing primary negative emotions. This insight could be vital for understanding FNDs manifestations and emphasizes the importance of considering emotional types and hemispheric lateralization in clinical practice. We found 47.6% left side hemiparesis in mimic compared to 33% in ischemic stroke.

The study also points to the need for a comprehensive understanding of SM especially in acute settings where rapid diagnosis is essential. Recognizing the potential psychosomatic origin of symptoms, particularly hemiparesis, and understanding the frequency of thrombolysis administration, underscores the necessity of a multidisciplinary approach for optimal patient care (8).

FNDs show a prevalence of 50 per 100,000 people and an incidence of 4–12 per 100,000 people per year (17, 18). Both sexes and all age groups are affected. In a study on psychogenic non-epileptic seizures, it was observed that the mean onset age for women was significantly lower than for men (19). Data from functional motor disorders also indicate a higher prevalence in female patients, most commonly presenting with weakness (43.9%) and tremor (40.7%) (20). Individuals with FMD typically have higher educational levels compared to the general population and frequently experience non-motor symptoms, especially anxiety, fatigue, and pain. Almost 50% of FNDs cases have an acute onset and require emergency service attention (21–23).

There is less data available about the incidence of SM due to FNDs in the emergency department. SM are increasing over time; they refer to a group of non-vascular conditions that present with neurological symptoms similar to those observed in acute strokes (24, 25).

Over the last decade, FNDs have been rising, accounting for 28–30% of all SM; notably, conversion disorder represents over 40% of psychogenic strokes (26).

Several predisposing factors, typically manifesting later in life, can influence the onset of FNDs. These include emotional and personality issues during adulthood and development. Precipitating factors, such as minor bodily injuries or panic episodes, occur closer to symptom onset (27). Perpetuating factors, which tend to extend hospitalization and illness duration, include a lack of acceptance of the diagnosis and other somatic symptom disorders (28).

In the recognition of FNDs, neurological examination can assist physicians, for example, in identifying positive signs. Joseph Babinski devoted much of his career to developing signs useful for distinguishing between organic and functional disorders (29, 30). The Hoover sign is one of the commonly described positive signs for detecting functional paresis in the lower limbs (31–33). While many other positive signs have been evaluated, diagnosing non-organic disease with certainty remains challenging (34–37).

New clinic protocols for identifying patients with SM have been proposed, such as the FAB score. This score considers advanced imaging for further diagnosis, and comprises variables: absence of facial droop, negative history of atrial fibrillation, age under 50 years, systolic blood pressure below 150 mm Hg at presentation, history of seizures, and isolated sensory symptoms without weakness at presentation (38).

EEG and hyper-acute imaging plays a fundamental role in the work-up of such patients. Identifying the site of vascular occlusion via CT angiography is mandatory; CT perfusion, using sequential scanning to detect changes in attenuation caused by the first pass of an iodinated contrast agent bolus, is crucial (26). However, clinical practice often cannot conclusively exclude a stroke diagnosis if the results are negative (39–43).

In acute settings, when the risk of missing a cerebrovascular disease is high, the potential benefits of intravenous thrombolysis often outweigh the potential harm of delayed treatment.

This study sheds light on the diagnostic and management challenges of SM cases within the FNDs population, providing valuable insights into the clinical characteristics and treatment patterns of these patients. It is crucial for improving the accuracy of stroke diagnosis and optimizing patient care. We also report, as previous data have indicated (9), that thrombolytic treatment is safe in selected cases.

### Highlights and limitations

FNDs mimicking a stroke represent a growing challenge in the ED. This study remarks the high incidence of SM due to FNDs in the acute setting and the safety profile of thrombolysis in those patients. There is little data in the literature on the management of FNDs in the emergency context, and little is still known about this type of disorder (are they a sort of panic attack?). We believe that a multidisciplinary approach and the use of advanced neuroimaging can help in the management of these cases. This analysis highlights the importance of demographic factors (age, gender, retirement status), clinical features (psychiatric disorders, language disturbances, visual disturbances), and medical history (stroke risk factors) in the identification of FNDs as stroke mimics.

This study also has several limitations, a first limitation is represented by the small number of the sample which therefore makes a precise statistical analysis difficult; furthermore it was difficult to compare the symptoms of SM due to FNDs with those of stroke patients, making the application of the Bamford classification complex for FNDs (44). The analysis of the stroke dataset also revealed several strengths and weaknesses in the approach and methods used. A significant strength is the comprehensive inclusion of variables related to demographics, medical history, and clinical features, which provided a robust basis for distinguishing between SM due to FNDs and Ischemic stroke cases. However, the weakness in the analyses is regarding the missing values. This issue pertains to education levels and occupational status. For this reason, in the analysis was applied the Iterative Imputer to the dataset with missing values. This technique allowed for the generation of a complete dataset, which was then used to perform logistic regression.

Future research could explore the integration of additional variables or alternative modeling approaches to further improve classification performance and provide more nuanced insights into stroke diagnosis.

## Data availability statement

The raw data supporting the conclusions of this article will be made available by the authors, without undue reservation.

## Ethics statement

‘The studies involving human participants were reviewed and approved by the Regional Ethics Committee of Friuli Venezia Giulia (CEUR FVG; Approval numbers: 115/2018 and 039_2020H). Written informed consent from the patients/participants or patients/participants’ legal guardian/next of kin was not required to participate in this study in accordance with the national legislation and the institutional requirements.

## Author contributions

PC: Conceptualization, Data curation, Funding acquisition, Methodology, Validation, Visualization, Writing – original draft. YR: Conceptualization, Data curation, Formal analysis, Validation, Visualization, Writing – original draft. LM: Data curation, Writing – review & editing. MQ: Data curation, Writing – review & editing. TL: Visualization, Writing – review & editing. SP: Visualization, Writing – review & editing. MC: Visualization, Writing – review & editing. AC: Visualization, Writing – review & editing. MB: Visualization, Writing – review & editing. AB: Supervision, Visualization, Writing – review & editing. PM: Funding acquisition, Validation, Visualization, Writing – review & editing.
